# Novel targeted therapies for central nervous system involvement in chronic lymphocytic leukemia: A systematic review

**DOI:** 10.1177/20406207261474934

**Published:** 2026-08-17

**Authors:** Abdulrahman F. Al-Mashdali, Mujahid O. Abdelraof, Raga M. Mukhtar, Shehab F. Mohamed

**Affiliations:** 1 Department of Hematology and Bone Marrow Transplant, National Center for Cancer Care and Research, 36977 Hamad Medical Corporation, Doha, Qatar; 2 Department of Public and Environmental Health, Faculty of Medicine, Gezira University, Wad Madani, Sudan; 3 Department of Physiology, 225782 International University of Africa, Khartoum, Sudan

**Keywords:** chronic lymphocytic leukemia, small lymphocytic lymphoma, central nervous system, BTK inhibitors, ibrutinib, venetoclax, targeted therapy

## Abstract

**Background:**

Central nervous system (CNS) involvement in chronic lymphocytic leukemia/small lymphocytic lymphoma (CLL/SLL) is rare, diagnostically challenging, and historically associated with poor outcomes. Bruton tyrosine kinase (BTK) and B-cell lymphoma 2 (BCL2) inhibitors may improve outcomes, but evidence remains limited to case reports and small series.

**Objectives:**

To summarize the clinical features, diagnostic findings, treatment patterns, efficacy, safety, and outcomes of CNS involvement by CLL/SLL treated with novel targeted therapies.

**Design:**

Systematic review of published case reports, case series, and cohorts with extractable individual-level data.

**Methods:**

PubMed, Scopus, and Web of Science were searched from database inception to September 15, 2025. Eligible studies reported adults with CNS involvement by CLL/SLL treated with targeted agents, including BTK inhibitors, venetoclax, duvelisib, dasatinib, or other novel therapies. Chemotherapy-only regimens and Richter transformation cases were excluded. Patient-level data were extracted, and study quality was assessed using the Murad tool.

**Results:**

Twenty-six studies including 32 patients were analyzed. Median age at CNS presentation was 65.5 years, and most cases occurred at relapse. CNS disease was meningeal in 47%, parenchymal in 22%, and combined in 31%. Adverse biological features, including TP53 mutation, del(17p), and unmutated immunoglobulin heavy chain variable region (IGHV), were common. Across 34 treatment regimens, the overall response rate was 94.1%, with 55.9% complete and 38.2% partial responses. Ibrutinib was the most frequently used agent, while venetoclax and ibrutinib–venetoclax showed favorable activity. Other targeted agents showed promising responses in isolated cases. Toxicities were infrequent and consistent with known safety profiles.

**Conclusion:**

BTK inhibitor- and venetoclax-based regimens show encouraging activity in CNS involvement by CLL/SLL, including in genomically adverse disease. However, the current evidence remains primarily exploratory and descriptive. Prospective multicenter studies are warranted to establish optimal treatment sequencing, combination strategies, and the role of adjunctive intrathecal therapy or radiotherapy.

## Introduction

Chronic lymphocytic leukemia(CLL) is the most common adult leukemia in Western countries^1^ and is often diagnosed incidentally, with many patients managed initially by active surveillance until treatment criteria are met, such as symptomatic lymphadenopathy, cytopenias from marrow failure, or disease-related symptoms.^2^Over the last decade, frontline therapy has shifted away from chemoimmunotherapy toward targeted agents that improve progression-free survival and, in some settings, overall survival. Oral Bruton Tyrosine Kinase (BTK) inhibitors such as ibrutinib and acalabrutinib, and more recently zanubrutinib, are standard options for many patients, including those with TP53 disruption or unmutated IGHV.^3^ Time-limited venetoclax-based combinations, especially venetoclax–obinutuzumab in treatment-naïve disease and venetoclax–rituximab in relapsed disease, induce deep remissions with high rates of undetectable MRD and allow fixed-duration therapy.Contemporary guidelines emphasize selecting therapy by biology and comorbidity, avoiding chemotherapy in TP53-aberrant disease, using BTK inhibitors when continuous oral therapy is preferred, and using venetoclax-based regimens when a finite course is desirable.^4^

Central nervous system(CNS) involvement in CLL is rare but clinically important.^5,6^ Large contemporary cohorts suggest that most neurologic symptoms in patients with CLL are due to alternative causes, and that truly clinically significant CNS CLL is uncommon; CSF testing is sensitive but has limited specificity, which creates a real risk of misclassification without careful clinical and imaging correlation. Strati et al. reported that other etiologies accounted for approximately 80 percent of neurologic presentations, underscoring the diagnostic challenge and the need for confirmatory approaches beyond CSF alone.^7^ Before the widespread adoption of targeted agents, management relied on intrathecal methotrexate or cytarabine, cranial or focal radiotherapy, and purine-analog–based systemic therapy. Outcomes in that era were variable and often poor, and the evidence base consisted largely of case reports and small series. Moazzam et al. synthesized pre-2010 literature and highlighted heterogeneity in diagnosis and treatment along with modest durability in many cases.^8^

The therapeutic landscape for CLL has since shifted. Ibrutinib and other BTK inhibitors produce deep and durable remissions systemically, and pharmacokinetic and clinical data indicate that ibrutinib reaches the CNS at biologically active concentrations in lymphomatous disease, providing a rationale for activity in CLL with CNS involvement.^9^ Likewise, venetoclax has been detected in human CSF at levels compatible with pharmacologic effect and has cleared CSF disease in CLL case reports, including after BTK inhibitor exposure.^10^ Despite these advances, there is still no consensus on diagnostic criteria or optimal therapy for CNS-involved CLL, and systematic descriptions remain scarce.

Against this backdrop, we conducted a systematic review of targeted agents in CNS CLL with the aim of characterizing patient demographics, cytogenetic features, and diagnostic approaches while also examining therapeutic strategies involving BTK inhibitors, venetoclax, and other novel agents. We assessed efficacy, safety, and follow-up outcomes, and explored potential predictors of response such as disease distribution and treatment regimen. By bringing together scattered case-level evidence from the novel-agent era and contrasting it with historical chemotherapy-based experience, this review provides much-needed perspective for clinicians managing suspected or confirmed CNS CLL.

## Methods

### Protocol and reporting

A protocol was developed a priori specifying eligibility criteria, outcomes, and analytic plans; it is available upon request. The review was conducted according to PRISMA 2020 guidelines for study identification, screening, and reporting.^11^

### Eligibility criteria

**Population.** Eligible studies included adults with chronic lymphocytic leukemia/small lymphocytic lymphoma (CLL/SLL) and central nervous system (CNS) involvement. CNS disease was accepted when confirmed by cerebrospinal fluid (CSF) cytology or flow cytometry, histopathologic confirmation from a CNS site, or neuroimaging findings consistent with CNS involvement by CLL/SLL in the appropriate clinical context. Reports describing Richter transformation involving the CNS were excluded.

**Interventions.** Eligible interventions included novel targeted therapies, including Bruton tyrosine kinase inhibitors such as ibrutinib, acalabrutinib, and zanubrutinib; venetoclax; duvelisib; dasatinib; and other reported targeted agents. Intrathecal therapy and radiotherapy were permitted as co-interventions. Reports describing conventional systemic chemotherapy alone, without a targeted agent, were excluded.

**Comparators.** A comparator group was not required.

**Outcomes.** Studies were eligible if they reported at least one extractable outcome, including neurologic, radiologic, or CSF response; overall clinical response; adverse events; survival; or follow-up status.

**Study design.** Eligible study designs included case reports, case series, and cohort studies with extractable individual-level data. Reviews, animal studies, abstracts without extractable outcomes, and duplicated cases were excluded. When overlapping reports were identified, the most complete or most recent report was retained.

**Time frame and language.** Studies published from database inception to the final search date were considered, with restriction to English-language publications.

### Information sources and search strategy

Comprehensive research was conducted in MEDLINE (via PubMed), Web of Science, and Scopus from database inception until the 15^th^ of September 2025. Search terms combined controlled vocabulary and free text for CLL, CNS involvement, leptomeningeal disease, brain, and targeted agents of interest. Grey literature was considered if individual-level data were extractable. Citation chaining and manual reference list screening supplemented database searches.

### Study selection

Two reviewers independently screened titles and abstracts, followed by full texts, against eligibility criteria. Discrepancies were resolved by consensus. When multiple reports described the same patient, data were consolidated from the most complete source. The study selection process is summarized in a PRISMA flow diagram.

### Data extraction

A piloted standardized form was used to capture study-level characteristics, patient demographics, diagnostic methods, molecular and cytogenetic features, treatment details, outcomes, and adverse events. Extraction was performed by one reviewer and independently verified by another. Narrative descriptions of outcomes were transcribed verbatim and mapped to standardized categories.

### Variable definitions

CNS involvement was classified as meningeal (including leptomeningeal, pachymeningeal, dural, or cranial nerve involvement), parenchymal (brain or spinal cord involvement), both, or uncertain. Treatment regimens were grouped as ibrutinib monotherapy, venetoclax monotherapy, or combined ibrutinib–venetoclax therapy, while other targeted agents were recorded individually. Responses were categorized as complete response (CR), partial response (PR), progression/no response, or not classifiable; responders were defined as patients achieving CR or PR.

Because response definitions were inconsistently reported across studies, standardized criteria were applied for pooled analysis. CR was defined as complete resolution of CNS disease based on the information provided in the original reports, including clinical, radiological, and/or cerebrospinal fluid (CSF) findings. PR was defined as clinical, radiological, or CSF improvement without complete resolution. CSF positivity required cytology or flow cytometry demonstrating a CLL clone, while MRI positivity was defined as reported imaging findings consistent with CNS involvement by CLL. Adverse events were categorized into bleeding, cardiac events, cytopenias, gastrointestinal toxicity, headache, and other reported toxicities.

### Risk of bias and study quality

Study quality was assessed using the Murad et al. tool for case reports and case series,^12^ which evaluates selection, ascertainment, causality, and reporting. Each study was rated as low, unclear, or high risk of bias. Assessments informed interpretation but did not determine inclusion.

### Data synthesis and analysis

All analyses were descriptive and conducted at the individual-patient level. Continuous variables were summarized as medians with interquartile ranges, while categorical variables were reported as counts and percentages using the number of available cases as denominators. Pooled summaries were generated for patient demographics, clinical presentation, diagnostic findings, genetic characteristics, treatments, and outcomes. Given the small sample sizes within treatment groups, heterogeneity in reporting, and the inherently case-based nature of the evidence, the findings should be interpreted descriptively rather than as confirmatory evidence. Accordingly, advanced statistical comparisons were neither feasible nor methodologically appropriate. Missing data were documented for each variable, and no imputation was performed.

## Results

### Search results

**Figure 1.**
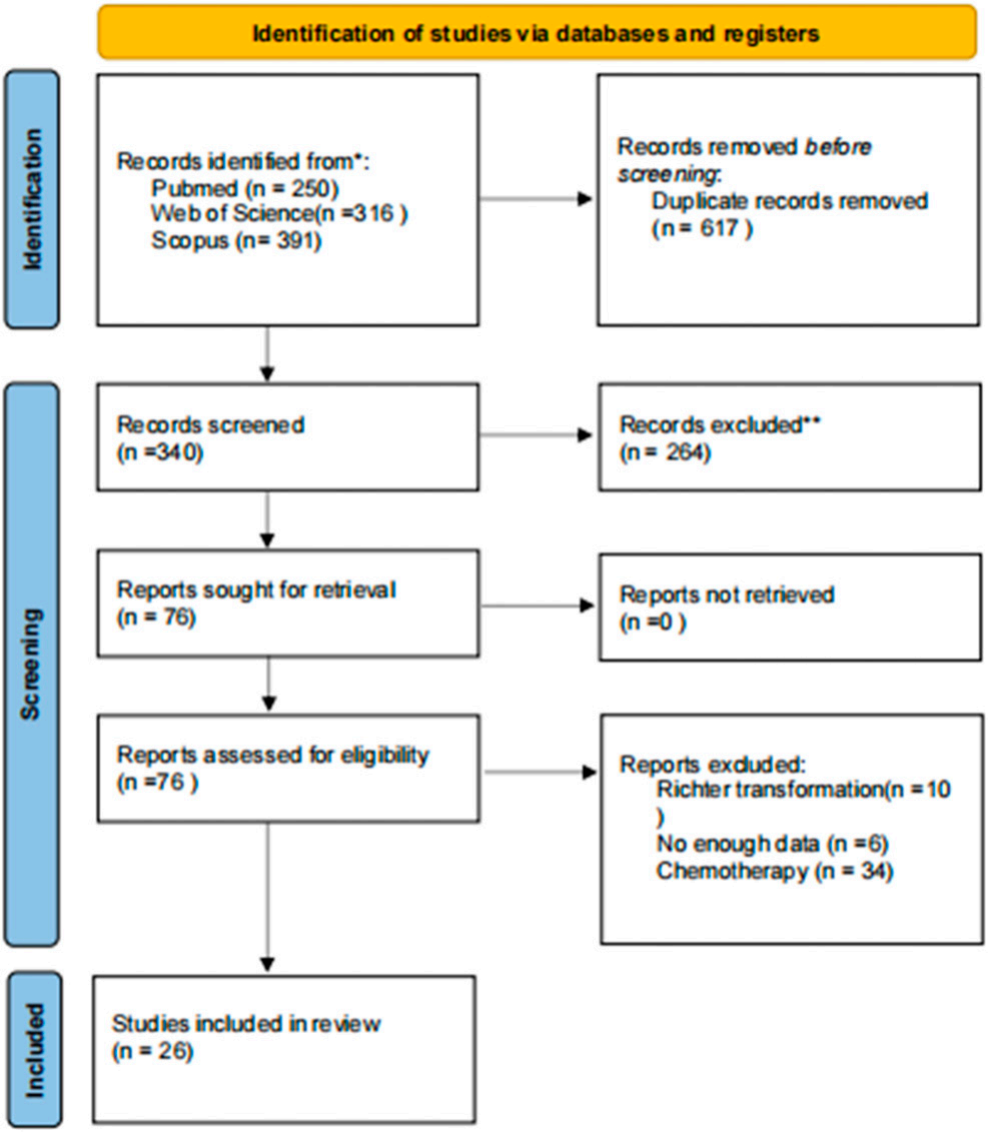
PRISMA flow diagram of the study selection process.

A comprehensive literature search was conducted across PubMed, Scopus, and Web of Science databases. The initial search retrieved (PubMed = 250, Scopus = 391, Web of Science = 316). After removal of duplicates, 340 unique records remained and were screened by title and abstract. Of these, 264 were excluded for not meeting the predefined inclusion criteria. The full texts of 76 articles were then assessed for eligibility, resulting in the inclusion of 26 studies (Published from 2010 to 2025) comprising a total of 32 patients with CLL with CNS involvement. The PRISMA flow diagram illustrates the study selection process in detail (Figure 1).

### Quality assessment

Using the Murad et al. methodological quality tool for case reports and case series, which covers selection, ascertainment, causality, and reporting, the included studies were judged overall at low risk of bias. Most reports clearly defined the cases, confirmed CNS involvement with objective methods such as CSF analysis, MRI, or histopathology, documented a plausible temporal relationship between targeted therapy and outcomes, and provided adequate follow up. Minor limitations included occasional incomplete histories of prior therapy, brief follow up in a few cases, and inconsistent use of standardized response terminology. These limitations were infrequent and unlikely to change the direction of the pooled findings (Supplemental 1).

### Study set and patient characteristics

We identified 32 published cases in 26 articles^5,6,10,13–35^ of CLL with CNS involvement treated with novel targeted agents, excluding chemotherapy. The median age at CNS presentation was 65.5 years (IQR 58.8–71.0; range 20–80). Most patients were male (24 of 32; 75.0%). CNS disease occurred at relapses in 21 cases (65.6%) and at first presentation in 11 (34.4%). Where available, the median interval from initial CLL diagnosis to CNS symptoms was 8.8 years (IQR 3.9–12.0; range 0.75–28.0; n=23). Baseline Rai or Binet staging was rarely reported but typically fell into the low-to intermediate-risk categories. White blood cell counts and LDH level were infrequently documented. The study set characteristics, genetic profiles, and diagnostic investigations of the included reports are summarized in Table 1.

**Table 1.** Study set characteristics, genetic profile, and diagnostic investigations.

Category	Metric/feature	Value/number	Percent
Demographics	Total cases	32	—
​	Median age at CNS presentation	65.5 years	—
​	Age range	20–80 years	—
​	Male	24	75.0%
​	Female	8	25.0%
Timing of CNS involvement	CNS involvement at relapse	21	65.6%
​	CNS involvement as first presentation of CLL/SLL	11	34.4%
​	Median interval from CLL diagnosis to CNS involvement	8.83 years	—
​	Interval range	0.75–28.0 years	—
Genomic features	Unmutated IGHV	7	—
​	TP53 mutation	6	—
​	del(17p)	6	—
​	del(11q)	2	—
​	Trisomy 12	8	—
​	Complex karyotype	6	—
Diagnostic findings	Positive CSF findings (among tested patients)	24/30	80.0%
​	MRI features of CNS involvement (among tested patients)	26/31	83.9%
​	CNS biopsy performed	7/32	21.9%

This table summarizes patient demographics, clinical presentation, genetic and molecular features, and main diagnostic investigations (CSF, MRI, and CNS biopsy). Percentages are calculated using the total cohort or relevant denominator as indicated.

### Genetic and molecular profile

Among 32 patients, trisomy 12 was the most frequently identified genetic abnormality, present in 8 cases. Unmutated IGHV status was observed in 7 patients, while TP53 mutation, del(17p), and complex karyotype were each identified in 6 patients. Del(11q) was less common, occurring in 2 patients (Table 1).

### Neurological manifestations

Symptoms varied by site of involvement. Visual complaints and cognitive or behavioral changes were each present in 6 patients (18.8%). Headache occurred in 5 (15.6%), motor weakness in 5 (15.6%), and gait ataxia or imbalance in 5 (15.6%). Cranial neuropathies were reported in 4 (12.5%). Sensory disturbances and back or neck pain occurred in 3 cases each (9.4%). Seizures and meningitic symptoms were each reported in 2 (6.2%). Autonomic or sphincter dysfunction and vertigo were each described once (3.1%).

### Patterns of CNS involvement and diagnostic confirmation

CNS involvement was meningeal in 15 patients (46.9%), parenchymal in 7 (21.9%), and combined in 10 (31.3%). Among those tested, CSF studies were positive in 24 of 30 (80.0%) and MRI was positive in 26 of 31 (83.9%). Biopsy confirmation of CLL involvement of CNS was performed in 7 cases (21.9%).

### Dose and dosing schedule details

Ibrutinib was most commonly administered at 420 mg once daily (12 reports), while a dose of 560 mg daily was reported in 5 cases.^5,19,28,32,35^ Venetoclax was generally administered at the standard dose of 400 mg daily (6 reports), except in the report by Beziat 2020,^34^ which used 200 mg daily. In the combination setting, Karbhari 2022^35^ used ibrutinib 560 mg plus venetoclax 400 mg with intrathecal therapy; Soumerai 2022^4^ used ibrutinib 420 mg plus venetoclax 400 mg without intrathecal therapy; and Reda 2019 ^10^ used venetoclax 400 mg daily with unspecified ibrutinib dosing and intrathecal chemotherapy.

### Treatments received and efficacy

**Figure 2.**
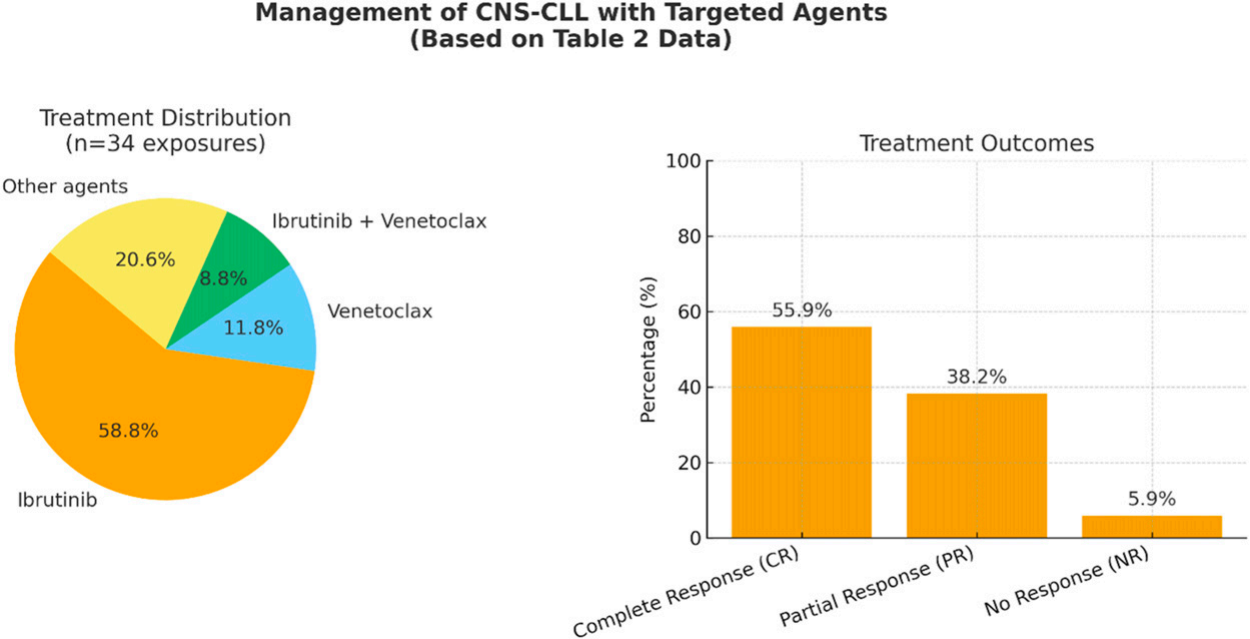
Distribution of targeted agents across 34 regimen-level exposures and corresponding treatment outcomes in CNS-CLL, showing predominance of ibrutinib-based therapy and high overall response rates.

Sixteen patients (50.0%) had not received systemic therapy before CNS involvement, reflecting a watch-and-wait strategy and most of them were asymptomatic and had no indication for treatment. Regarding the treatment of CNS involvement in CLL, a total of 34 treatment encounters were recorded across 32 patients, as two individuals received two distinct novel agents sequentially after CNS involvement. These therapies were administered at separate timepoints and produced independent clinical outcomes. To accurately reflect treatment-level efficacy, the analysis was performed at the regimen level (n = 34), not per patient. The novel agents used and treatment responses across the reports included are summarized in Table 2 and Figure 2.

**Table 2.** Novel agents used and treatment response.

Novel regimen	N	CR	PR	NR	ORR (%)
Ibrutinib alone	20	11	8	1	95.0
Venetoclax alone	4	2	2	0	100.0
Ibrutinib + venetoclax	3	3	0	0	100.0
Acalabrutinib	3	1	2	0	100.0
Zanubrutinib	1	0	1	0	100.0
Dasatinib	1	1	0	0	100.0
Pomalidomide	1	1	0	0	100.0
Ibrutinib + duvelisib	1	0	0	1	0.0
Total	34	19	13	2	94.1

CR = complete response; PR = partial response; NR = no response. The table summarizes clinical outcomes for each novel regimen administered after CNS involvement, with results presented per treatment exposure (n=34) rather than per patient.

Overall, 19 of 34 treatment encounters (55.9%) achieved a complete response, 13 (38.2%) achieved a partial response, and 2 (5.9%) showed no response, yielding an overall response rate (ORR) of 94.1%.

Among the most used agents, ibrutinib monotherapy accounted for 20 of the 34 regimens, with 95.0% overall response. Venetoclax monotherapy produced 100% response across 4 patients, and combination ibrutinib plus venetoclax also achieved 100% response in 3 cases.

Acalabrutinib was used in three patients:

• Partial responses were reported in Chauhan 2020^28^ and Vicino 2023^14^ (in the latter, the patient had neurolymphomatosis confirmed by positive CSF and MRI and received acalabrutinib with obinutuzumab, leading to significant improvement).
• A complete response was described in Alshamali 2022.^13^

Other novel agents showed favorable outcomes:

• Zanubrutinib led to a partial response (Webb 2024^15^).
• Dasatinib resulted in a complete response (Russwurm 2010^16^).
• Pomalidomide, given in combination, induced a complete response (Qin 2024^5^).

### Treatment switching and dual outcomes

To avoid confusion regarding the treatment count (n=34 vs 32 patients), we highlight that two patients received sequential BTK inhibitors following CNS involvement, each producing distinct responses:

• In Alshamali 2022,^13^ the patient initially received acalabrutinib after CNS presentation. After relapse due to treatment interruption, they were switched to ibrutinib monotherapy upon CSF confirmation of relapse. Both regimens yielded clinical benefit.
• In Chauhan 2020,^28^ the patient was transitioned from ibrutinib to acalabrutinib due to intolerance (diarrhea and bruising). Remarkably, the patient experienced partial response to ibrutinib and acalabrutinib with signifcant improvement in vision acuity.

### Non-responders and progression

Two patients demonstrated frank disease progression despite intensive therapy:

• In Nakanashi 2020,^25^ the disease was refractory to high-dose methotrexate, whole-brain irradiation, FCR chemoimmunotherapy, and ibrutinib.
• In Shestakova 2025,^20^ patient number 2 died from progressive CNS CLL despite receiving triple intrathecal chemotherapy, ibrutinib, rituximab, and duvelisib. CSF studies remained positive throughout.

### Safety

Most reports documented no treatment-limiting toxicity. Recorded events included gastrointestinal toxicity (3 cases: Chauhan 2020, Qin 2024, Webb 2024^5,15,28^), bleeding or bruising (3 cases: Chauhan 2020, Qin 2024, Webb 2024^5,15,28^), cytopenias (2 cases: Beziat 2020, Webb 2024^15,34^), headache (1 case: Karbhari 2022^35^), and a cardiac event with atrial fibrillation and stroke (1 case: Wanquet 2016^18^). Overall tolerability of targeted agents in CNS disease was favorable.

### Follow-up and outcomes

Several cases described sustained neurologic and radiographic remission exceeding one year, including reports by Reda 2019, Dewaide 2023, Evans 2022, and Tam 2016.^10,17,23,31^

### Exploratory predictors of response

High response rates were observed even in patients with adverse biological features. Cases with del(17p), TP53 mutations, or unmutated IGHV achieved complete or partial remissions under targeted therapy, underscoring the ability of BTK inhibitors and venetoclax to overcome traditionally poor prognostic markers. Importantly, meningeal disease showed a higher proportion of complete responses compared with parenchymal or combined sites, though response rates across all disease patterns remained encouraging. Diagnostic modality (CSF, MRI, or biopsy) did not appear to segregate outcomes, suggesting that disease biology and treatment choice may be more decisive. Also, regarding first presentation versus relapse, outcomes were favorable in both groups, but relapsed CNS cases achieved a higher proportion of complete response compared with first-presentation cases.

When stratified by treatment, ibrutinib- and venetoclax-based regimens consistently induced durable remissions, and every reported case of ibrutinib plus venetoclax achieved complete response, pointing to a potential synergistic effect. Other novel agents (e.g., acalabrutinib, dasatinib, zanubrutinib, pomalidomide) showed variable but promising results, often in relapsed or multiply pretreated patients. Exploratory predictors of response in CLL with CNS involvement are summarized in Table 3.

**Table 3.** Exploratory predictors of response in CLL with CNS involvement.

Category	Subgroup	CR	PR	NR	Total
Poor-risk biology	No	10 (52.6%)	8 (42.1%)	1 (5.3%)	19
Poor-risk biology	Yes	8 (61.5%)	4 (30.8%)	1 (7.7%)	13
Disease site	Meningeal	8 (53.3%)	6 (40.0%)	1 (6.7%)	15
Disease site	Parenchymal	2 (28.6%)	4 (57.1%)	1 (14.3%)	7
Disease site	Both	8 (80.0%)	2 (20.0%)	0 (0.0%)	10
Presentation status	First presentation	5 (45.5%)	5 (45.5%)	1 (9.1%)	11
Presentation status	Relapse	13 (61.9%)	7 (33.3%)	1 (4.8%)	21
Treatment	Ibrutinib	10 (50.0%)	8 (40.0%)	2 (10.0%)	20
Treatment	Ibrutinib + venetoclax	3 (100.0%)	0 (0.0%)	0 (0.0%)	3
Treatment	Other novel agents	2 (50.0%)	2 (50.0%)	0 (0.0%)	4
Treatment	Venetoclax	2 (50.0%)	2 (50.0%)	0 (0.0%)	4

Table summarizes treatment responses according to biological risk factors, disease site, presentation status, and treatment type. Numbers represent absolute counts with percentages in parentheses. CR = Complete Response, PR = Partial Response, NR = No Response.

These findings suggest that in CLL with CNS involvement, poor-risk genetic features do not preclude efficacy of targeted therapy, meningeal disease may be particularly sensitive, and combination BTK–BCL2 inhibition deserves further exploration.

## Discussion

In the pre-targeted therapy era, CNS involvement in CLL was almost invariably considered a fatal or refractory event. Reports from the 1990s and early 2000s often described interventions with high-dose methotrexate, intrathecal chemotherapy, or cranial irradiation, yet these approaches typically produced only temporary neurologic relief before progression recurred.^36^ Fludarabine-based regimens, although effective in systemic CLL, offered little benefit in the CNS setting and were frequently limited by toxicity in older, comorbid patients. In a retrospective study by Strati and colleagues,^7^ patients with clinically significant CNS involvement had a median overall survival of just 12 months despite receiving therapy, findings that aligned with other series reporting similarly poor outcomes. These sobering results reflected two major barriers: first, the poor penetration of conventional cytotoxic drugs across the blood–brain barrier, and second, the indolent biology of CLL itself, which often blunted the impact of intensive chemotherapy while amplifying treatment-related morbidity. As a result, CNS-CLL was historically regarded as a complication with little chance of durable remission, and most management strategies were primarily palliative rather than curative.

By contrast, our review of novel-agent–treated CNS-CLL suggests a more favorable therapeutic signal. We compiled 32 cases (34 regimen-level observations), and observed an overall response rate of 94.1%, with 55.9% achieving complete remission and the rest partial responses. These rates far exceed what could reasonably be expected from chemotherapy-era approaches. Notably, even among patients harboring adverse biological features including del(17p), TP53 mutations, or unmutated IGHV significant remissions were achieved. That suggests that modern agents might overcome the biological disadvantages that chemotherapy could not, consistent with landmark trials done in high-risk group treated with ibrutinib and other BTKi.^37^

These observations support the biologic plausibility that modern small molecules can penetrate the CNS at therapeutically relevant levels. In studies of primary CNS lymphoma, ibrutinib concentrations in CSF have been shown to exceed the in vitro IC_50_ for malignant B cells, and patients experienced rapid neurologic improvement, demonstrating that ibrutinib reaches the CNS in clinically meaningful amounts.^9^ Preclinical CNS lymphoma models further confirmed this finding, as ibrutinib achieved high concentrations in both brain tissue and CSF and significantly prolonged survival compared with controls.^38^In a phase II clinical trial evaluating ibrutinib in CNS lymphoma, approximately two-thirds of patients achieved an objective response, although the durability of these responses was typically measured in months rather than years.^39^Of note, many reports in our review administered ibrutinib at 420 mg daily, which is lower than the doses typically recommended in CNS lymphoma (560–840 mg). Interestingly, patients still achieved meaningful responses at this reduced dose, suggesting that effective CNS activity may be attainable even below the higher dosing used in lymphoma.^40^

Similarly, venetoclax has been directly measured in CSF in patients with hematologic malignancies. In a report of CLL with CNS involvement, venetoclax was detected in the CSF at low-nanogram concentrations consistent with pharmacologic activity and was associated with clearance of CSF disease following prior BTK inhibitor failure.^10^In a broader pharmacokinetic study of leukemia patients, venetoclax concentrations in CSF ranged from <0.1 to ∼11 ng/mL, with a mean level of approximately 2.8 ng/mL and a plasma-to-CSF ratio near 300.^41^Although these concentrations are modest compared with plasma levels, they fall within a range associated with biologic activity in preclinical models and may be sufficient to achieve therapeutic effects in the relatively low-volume CNS disease compartment.

Long-term follow-up from the RESONATE-2 and Alliance A041202 trials demonstrated durable efficacy of ibrutinib monotherapy in CLL, including among patients with high-risk genomic features such as unmutated IGHV, del(17p), TP53 mutation, and complex karyotype.^42,43^ Our pooled CNS-CLL analysis parallels these systemic findings, with clinical responses observed even in patients with adverse-risk biology and several reports describing durable remissions in heavily pretreated cases. Venetoclax monotherapy has likewise demonstrated substantial activity in relapsed and high-risk CLL, including in patients with del(17p) and TP53 aberrations, while combination therapy with ibrutinib and venetoclax has produced deep and durable remissions across high-risk subgroups^44,45^ . In our pooled CNS-CLL cohort, favorable responses were reported with both BTK inhibitor and venetoclax monotherapy, as well as with combined ibrutinib–venetoclax therapy, including several complete remissions. Although these observations are based on limited case-level evidence and may be influenced by publication bias, they suggest that BTK and BCL2 inhibition may retain activity in CNS-CLL. Safety in our pooled cases largely matched known class profiles. For BTK inhibitors, bleeding and atrial fibrillation are the key concerns and can require interruption or switching agents. For venetoclax, tumor lysis risk mandates prophylaxis and staged ramp-up; case reports and clinical reviews reiterate that careful monitoring allows safe delivery even in heavily pretreated patients. Importantly, our case-based synthesis likely underestimates toxicity due to publication bias toward favorable stories.

Recently, the ERIC group reported an international retrospective study of 48 patients with CNS involvement by CLL, representing the largest multicenter cohort published to date. Among 47 treated patients, approximately half received targeted agents, most commonly a Bruton tyrosine kinase inhibitor (BTKi), whereas 34% received chemoimmunotherapy. Initial therapy was associated with high rates of clinical and radiologic improvement, including reduction or complete resolution of neurologic symptoms and imaging abnormalities in most patients. The estimated 5-year overall survival from CNS involvement was 77.1%, and the 5-year time to next treatment or death was higher among patients treated with BTKi than among those treated with chemoimmunotherapy. Attainment of CNS complete response after initial therapy was also associated with longer overall survival. Although the ERIC study was published after our final search date and manuscript submission and reported aggregate, study-level data from a mixed-treatment cohort, its findings provide important external support for the emerging role of BTKi-based therapy and complement our patient-level synthesis of targeted therapy outcomes in published CNS-CLL cases^46^.

### Limitations

This review should be interpreted with caution. The available evidence consists mainly of case reports and small case series, which are inherently susceptible to selection bias, publication bias, incomplete reporting, and short or inconsistent follow-up. Publication bias is particularly important, as successful or unusual responses to novel targeted therapies are more likely to be reported, whereas treatment failures, toxicities, or less favorable outcomes may be underrepresented. Although the Murad tool indicated an overall low risk of bias among the included reports, heterogeneity in diagnostic methods, co-interventions, treatment sequencing, and response definitions limits the strength of pooled conclusions.

Accordingly, the treatment-related findings should be considered descriptive and hypothesis-generating rather than comparative or definitive. The small number of patients within each treatment subgroup precluded robust statistical comparisons, and apparent differences between regimens, disease sites, or biological risk groups may reflect case selection, reporting heterogeneity, or publication bias rather than true efficacy differences.

Despite these limitations, this review provides a systematic patient-level synthesis of a rare manifestation of CLL/SLL and identifies clinically relevant patterns across modern targeted therapies. However, the apparent frequent responses to BTK inhibitors and venetoclax-based regimens, as well as the uncertain role of intrathecal therapy, should be interpreted cautiously because of non-standardized treatment allocation, frequent concurrent systemic therapy, and potential underreporting of unfavorable outcomes.

### Future directions

Prospective multicenter registries are needed to move beyond anecdotal evidence in CNS involvement by CLL. Future studies should use standardized diagnostic criteria, CNS-specific response definitions, uniform treatment documentation, and longer follow-up to clarify the optimal sequencing and combination of BTK inhibitors, venetoclax, intrathecal therapy, and radiotherapy. Incorporating CSF pharmacokinetic sampling and stratification by anatomic pattern, TP53 aberrations, IGHV status, and prior therapy may help determine whether observed differences in response, particularly in meningeal disease, reflect true treatment sensitivity or case mix. Pragmatic studies comparing BTK inhibitor monotherapy with BTK plus venetoclax are especially warranted. Additionally, bispecific antibodies may represent a future therapeutic direction for CNS lymphoid disease. A recent report described the use of glofitamab in Richter transformation with isolated CNS involvement^47^ however, Richter transformation is biologically and clinically distinct from CLL/SLL and was excluded from the present review according to the predefined eligibility criteria. Currently, published data on bispecific antibodies specifically for CNS involvement by CLL/SLL without Richter transformation are lacking, and further investigation is warranted.

## Conclusion

This systematic review suggests that BTK inhibitors and venetoclax are associated with favorable clinical responses in patients with CNS-CLL, including some with adverse genomic features. Combination regimens may offer additional benefit, and encouraging activity was also observed with second-generation BTK inhibitors. However, these observations are derived primarily from case reports and small case series and may be influenced by publication bias and selective reporting of favorable outcomes. Although the available evidence suggests that novel agents may address some limitations associated with chemotherapy-era approaches, prospective multicenter studies are needed to better define treatment durability, optimize diagnostic strategies, and inform therapeutic sequencing in this challenging clinical setting.

## Supplemental material

Supplemental material - Novel targeted therapies for central nervous system involvement in chronic lymphocytic leukemia: A systematic review Supplemental material for Novel targeted therapies for central nervous system involvement in chronic lymphocytic leukemia: A systematic review by Abdulrahman F. Al-Mashdali, Mujahid O. Abdelraof, Raga M. Mukhtar, Shehab F. Mohamed in Therapeutic Advances in Hematology.

## Data Availability

All data analyzed in this study were extracted from published articles included in the systematic review. The extracted dataset is available from the corresponding author upon reasonable request.*
